# Supplementary figures and images for: Self-assembly of pericentriolar material in interphase cells lacking centrioles
Source: eLife. 2022 Jul 5;11:e77892. doi: 10.7554/eLife.77892 (PMC9307276; doi:10.7554/eLife.77892)

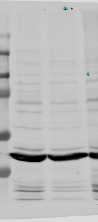

Supplement: Figure 2—figure supplement 1—source data 1. [file elife-77892-fig2-figsupp1-data1.zip › panel A/2KO, with and without CB, anti Actin.png]

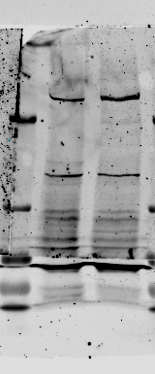

Supplement: Figure 2—figure supplement 1—source data 1. [file elife-77892-fig2-figsupp1-data1.zip › panel A/2KO, with and without CB, anti CEP192 and anti Ku80.png]

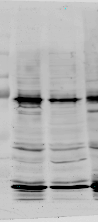

Supplement: Figure 2—figure supplement 1—source data 1. [file elife-77892-fig2-figsupp1-data1.zip › panel A/2KO, with and without CB, anti NEDD1.png]

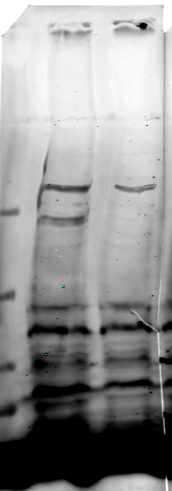

Supplement: Figure 2—figure supplement 1—source data 1. [file elife-77892-fig2-figsupp1-data1.zip › panel B/siCDK5RAP2/2KO+CB, siCDK5RAP2, anti CDK5RAP2, anti GM130.png]

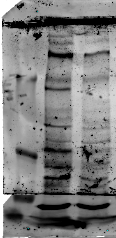

Supplement: Figure 2—figure supplement 1—source data 1. [file elife-77892-fig2-figsupp1-data1.zip › panel B/siCEP152/2KO+CB, siCEP152, anti CEP152, anti Ku80.png]

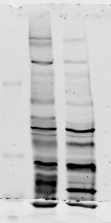

Supplement: Figure 2—figure supplement 1—source data 1. [file elife-77892-fig2-figsupp1-data1.zip › panel B/siCEP192/2KO+CB, siCEP192, anti CEP192, anti Ku80.png]

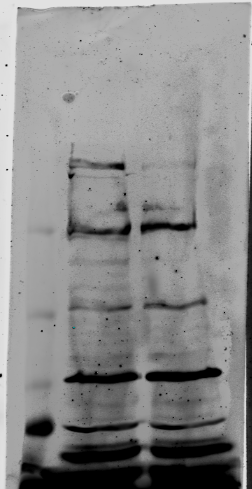

Supplement: Figure 2—figure supplement 1—source data 1. [file elife-77892-fig2-figsupp1-data1.zip › panel B/siDHC/2KO+CB, siDHC, anti DHC.png]

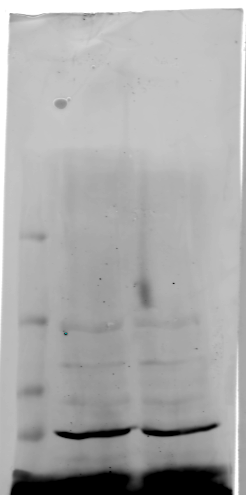

Supplement: Figure 2—figure supplement 1—source data 1. [file elife-77892-fig2-figsupp1-data1.zip › panel B/siDHC/2KO+CB, siDHC, anti Ku80.png]

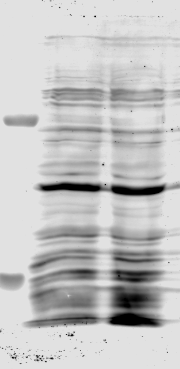

Supplement: Figure 2—figure supplement 1—source data 1. [file elife-77892-fig2-figsupp1-data1.zip › panel B/sigamma-tubulin/2KO+CB, siGamma-tubulin, anti beta-tubulin.png]

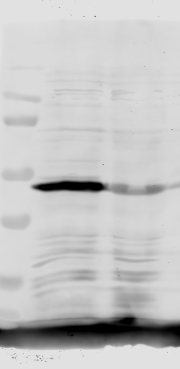

Supplement: Figure 2—figure supplement 1—source data 1. [file elife-77892-fig2-figsupp1-data1.zip › panel B/sigamma-tubulin/2KO+CB, siGamma-tubulin, anti gamma-tubulin.png]

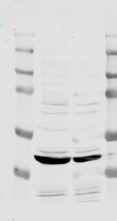

Supplement: Figure 2—figure supplement 1—source data 1. [file elife-77892-fig2-figsupp1-data1.zip › panel B/siNEDD1/2KO+CB, siNEDD1, anti Actin.png]

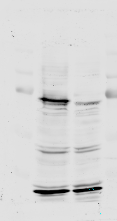

Supplement: Figure 2—figure supplement 1—source data 1. [file elife-77892-fig2-figsupp1-data1.zip › panel B/siNEDD1/2KO+CB, siNEDD1, anti NEDD1.png]

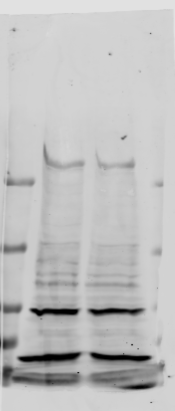

Supplement: Figure 2—figure supplement 1—source data 1. [file elife-77892-fig2-figsupp1-data1.zip › panel B/siNIN/2KO+CB, siNIN, anti BICD2.png]

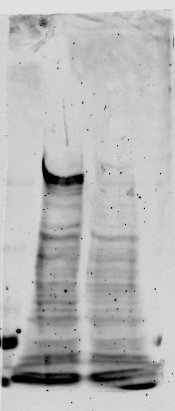

Supplement: Figure 2—figure supplement 1—source data 1. [file elife-77892-fig2-figsupp1-data1.zip › panel B/siNIN/2KO+CB, siNIN, anti NIN.png]

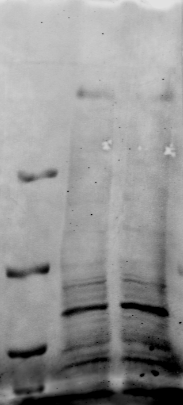

Supplement: Figure 2—figure supplement 1—source data 1. [file elife-77892-fig2-figsupp1-data1.zip › panel B/siPCNT/2KO+CB, siPCNT, anti GM130.png]

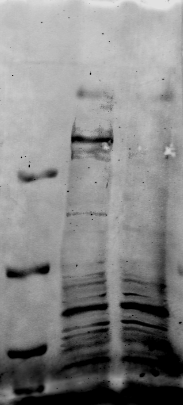

Supplement: Figure 2—figure supplement 1—source data 1. [file elife-77892-fig2-figsupp1-data1.zip › panel B/siPCNT/2KO+CB, siPCNT, anti PCNT.png]

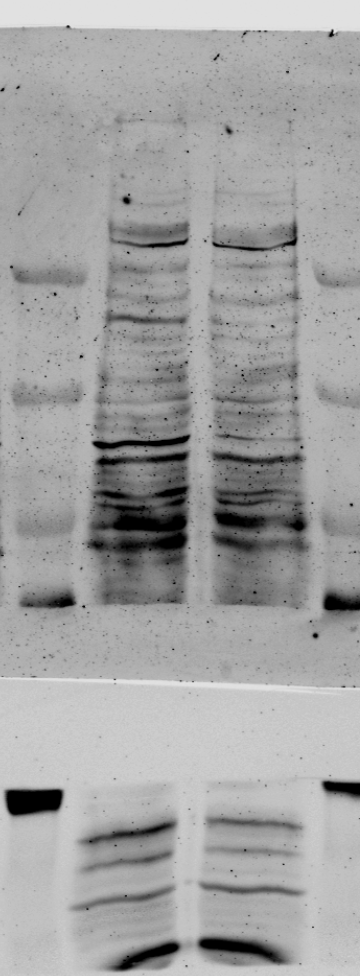

Supplement: Figure 2—figure supplement 2—source data 1. [file elife-77892-fig2-figsupp2-data1.zip › panel F/AKAP450-CAMSAP2 KO, AKAP450-CAMSAP2-p53 KO, anti Actin.png]

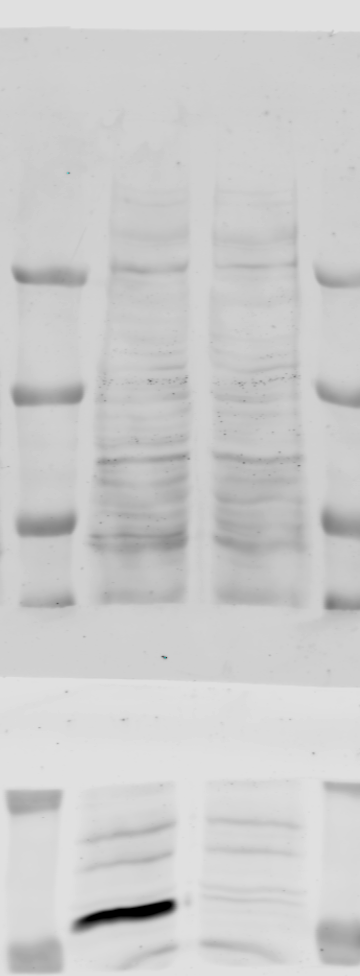

Supplement: Figure 2—figure supplement 2—source data 1. [file elife-77892-fig2-figsupp2-data1.zip › panel F/AKAP450-CAMSAP2 KO, AKAP450-CAMSAP2-p53 KO, anti p53.png]

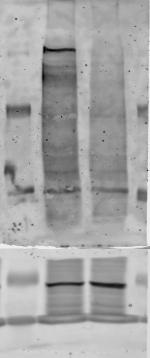

Supplement: Figure 2—figure supplement 2—source data 1. [file elife-77892-fig2-figsupp2-data1.zip › panel K/AKAP450-CAMSAP2-p53 KO, AKAP450-CAMSAP2-p53-PCNT KO, anti PCNT, anti Ku80.png]

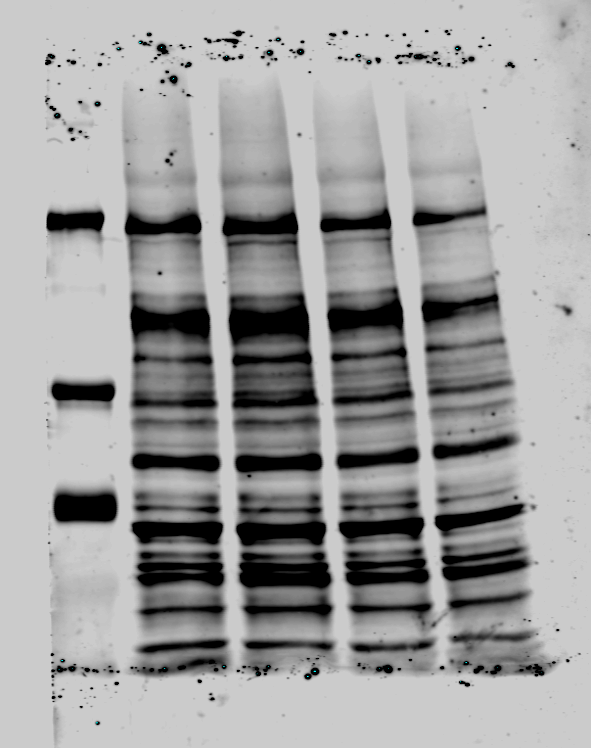

Supplement: Figure 2—figure supplement 4—source data 1. [file elife-77892-fig2-figsupp4-data1.zip › AKAP450-CAMSAP2-p53 KO, AKAP450-CAMSAP2-p53-PCNT KO, with or without CentB treatment, anti CDK5RAP2, anti Ku80.png]

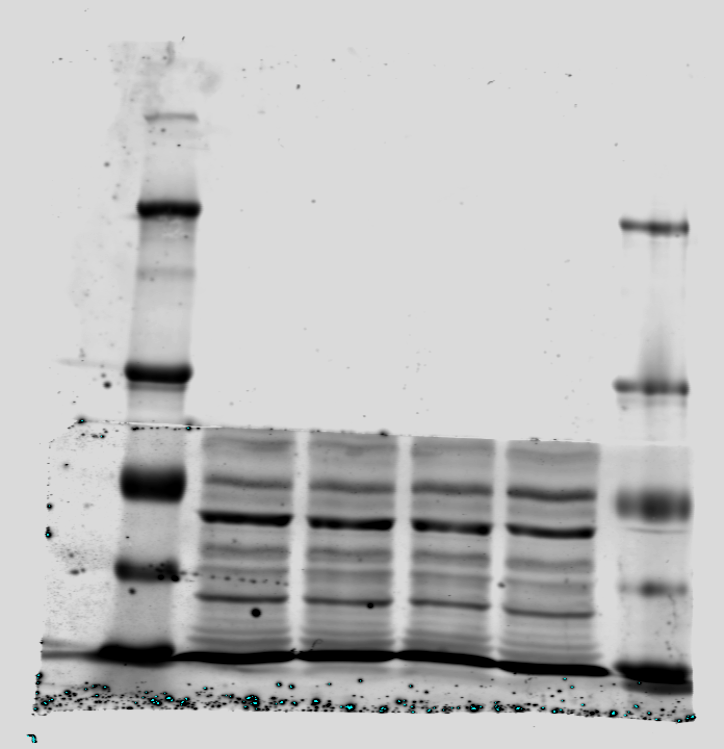

Supplement: Figure 2—figure supplement 4—source data 1. [file elife-77892-fig2-figsupp4-data1.zip › AKAP450-CAMSAP2-p53 KO, AKAP450-CAMSAP2-p53-PCNT KO, with or without CentB treatment, anti Ku80, anti gamma-tubulin.png]

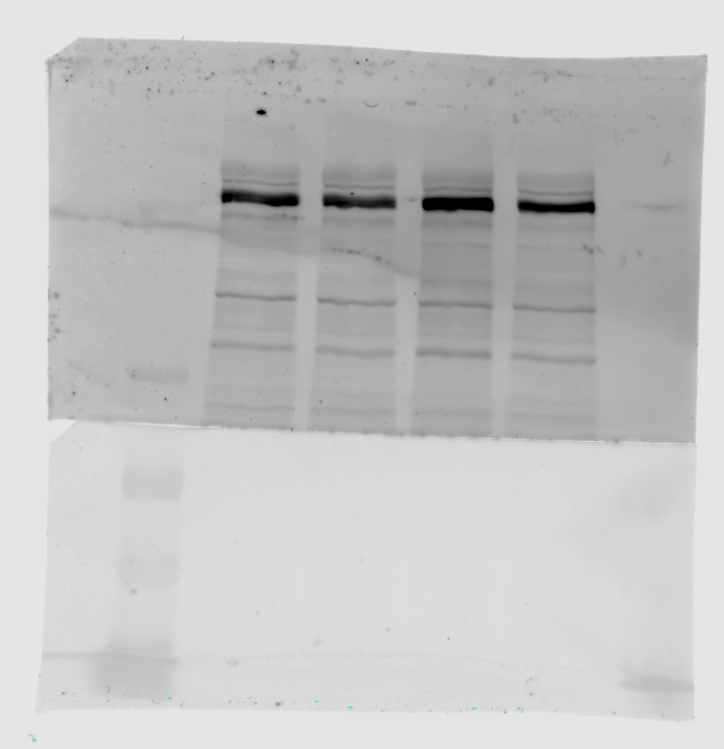

Supplement: Figure 2—figure supplement 4—source data 1. [file elife-77892-fig2-figsupp4-data1.zip › AKAP450-CAMSAP2-p53 KO, AKAP450-CAMSAP2-p53-PCNT KO, with or without CentB treatment, anti NIN.png]

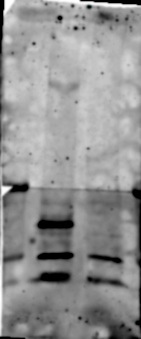

Supplement: Figure 2—figure supplement 5—source data 1. [file elife-77892-fig2-figsupp5-data1.zip › EB1,EB3 mut, AKAP450-CAMSAP2-EB1-EB3 mut, anti AKAP450, anti CAMSAP2, short exposure.png]

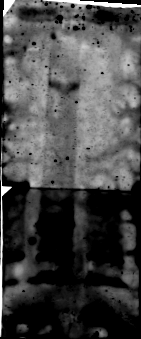

Supplement: Figure 2—figure supplement 5—source data 1. [file elife-77892-fig2-figsupp5-data1.zip › EB1,EB3 mut, AKAP450-CAMSAP2-EB1-EB3 mut, anti AKAP450, anti CAMSAP2, long exposure.png]

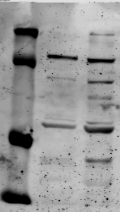

Supplement: Figure 2—figure supplement 5—source data 1. [file elife-77892-fig2-figsupp5-data1.zip › EB1,EB3 mut, AKAP450-CAMSAP2-EB1-EB3 mut, anti Ku80.png]

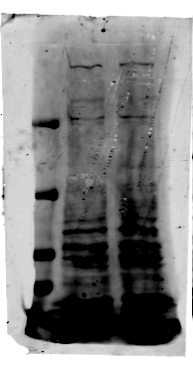

Supplement: Figure 4—source data 2. [file elife-77892-fig4-data2.zip › AKAP450-CAMSAP2 KO + CentB, with and without Dynaprazole treatment, anti DHC.png]

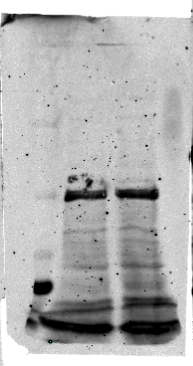

Supplement: Figure 4—source data 2. [file elife-77892-fig4-data2.zip › AKAP450-CAMSAP2 KO + CentB, with and without Dynaprazole treatment, anti p150Glued.png]

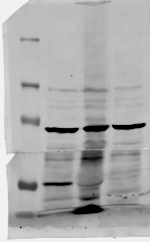

Supplement: Figure 7—figure supplement 3—source data 1. [file elife-77892-fig7-figsupp3-data1.zip › AKAP450-CAMSAP2 KO, AKAP450-CAMSAP2-p53-PCNT KO, AKAP450-CAMSAP2-CDK5RAP2-MMG-p53-PCNT KO, anti Ku80, anti p53.png]

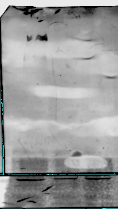

Supplement: Figure 7—figure supplement 3—source data 1. [file elife-77892-fig7-figsupp3-data1.zip › AKAP450-CAMSAP2 KO, AKAP450-CAMSAP2-p53-PCNT KO, AKAP450-CAMSAP2-CDK5RAP2-MMG-p53-PCNT KO, anti PCNT.png]

Source data 1  
Uncropped Western blots shown in this manuscript

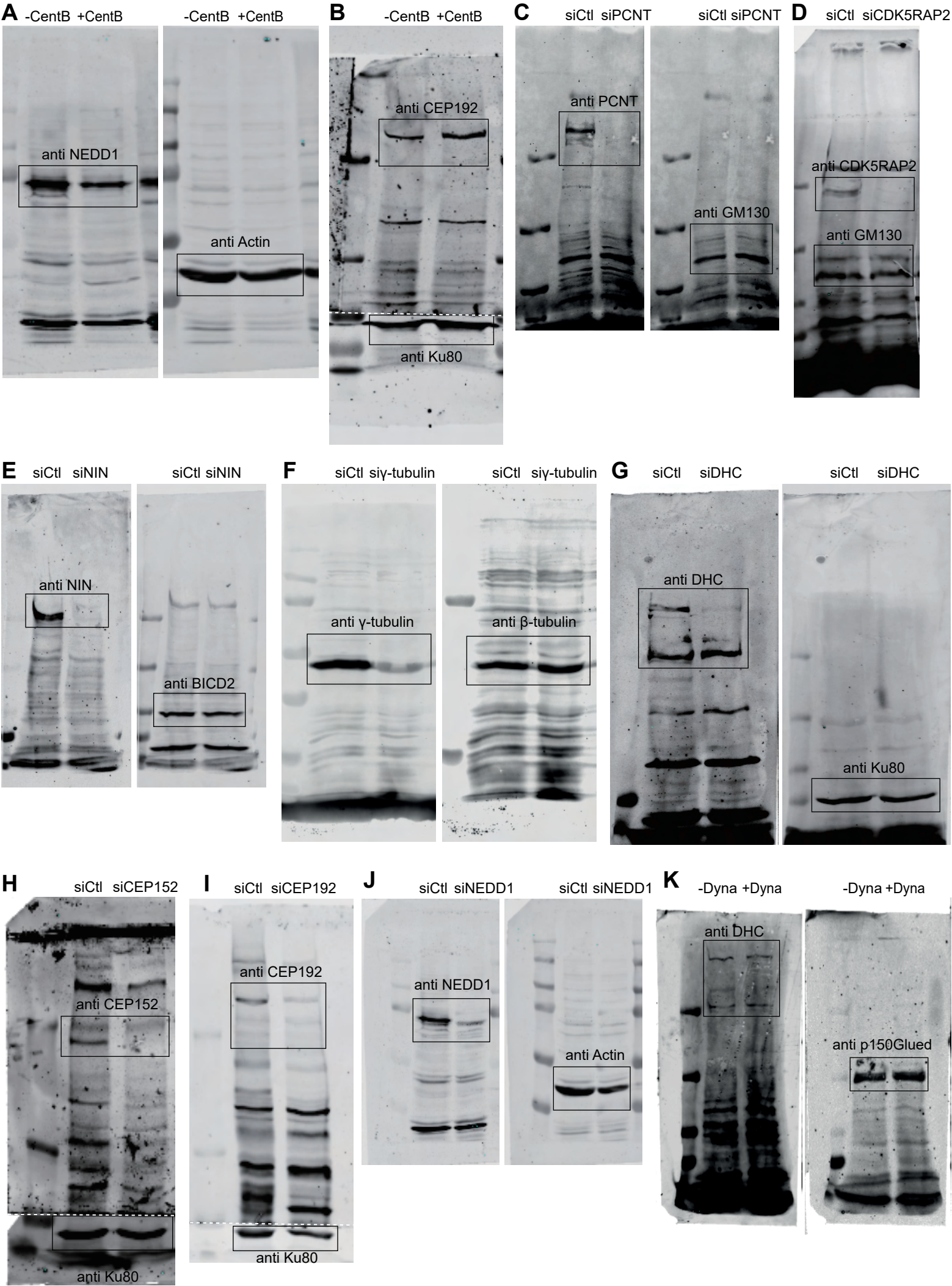

Supplement: Source data 1. — (A,B) Western blots showing that NEDD1 and CEP192 are present in centrinone-treated AKAP450/CAMSAP2 knockout cells shown in Figure 2—figure supplement 1A. (C-J) Western blots showing the depletion of indicated proteins in centrinone-treated AKAP450/CAMSAP2 knockout cells shown in Figure 2—figure supplement 1B. White dashed lines indicate where the blots were cut before incubation. (K) Western blot showing that 3 hrs treatment with dynapyrazole A does not affect the expression of the endogenous dynein heavy chain and the dynactin large subunit p150Glued in centrinone-treated AKAP450/CAMSAP2 knockout cells shown in Figure 4D. [file elife-77892-data1.pdf]

Source data 2  
Uncropped Western blots shown in this manuscript

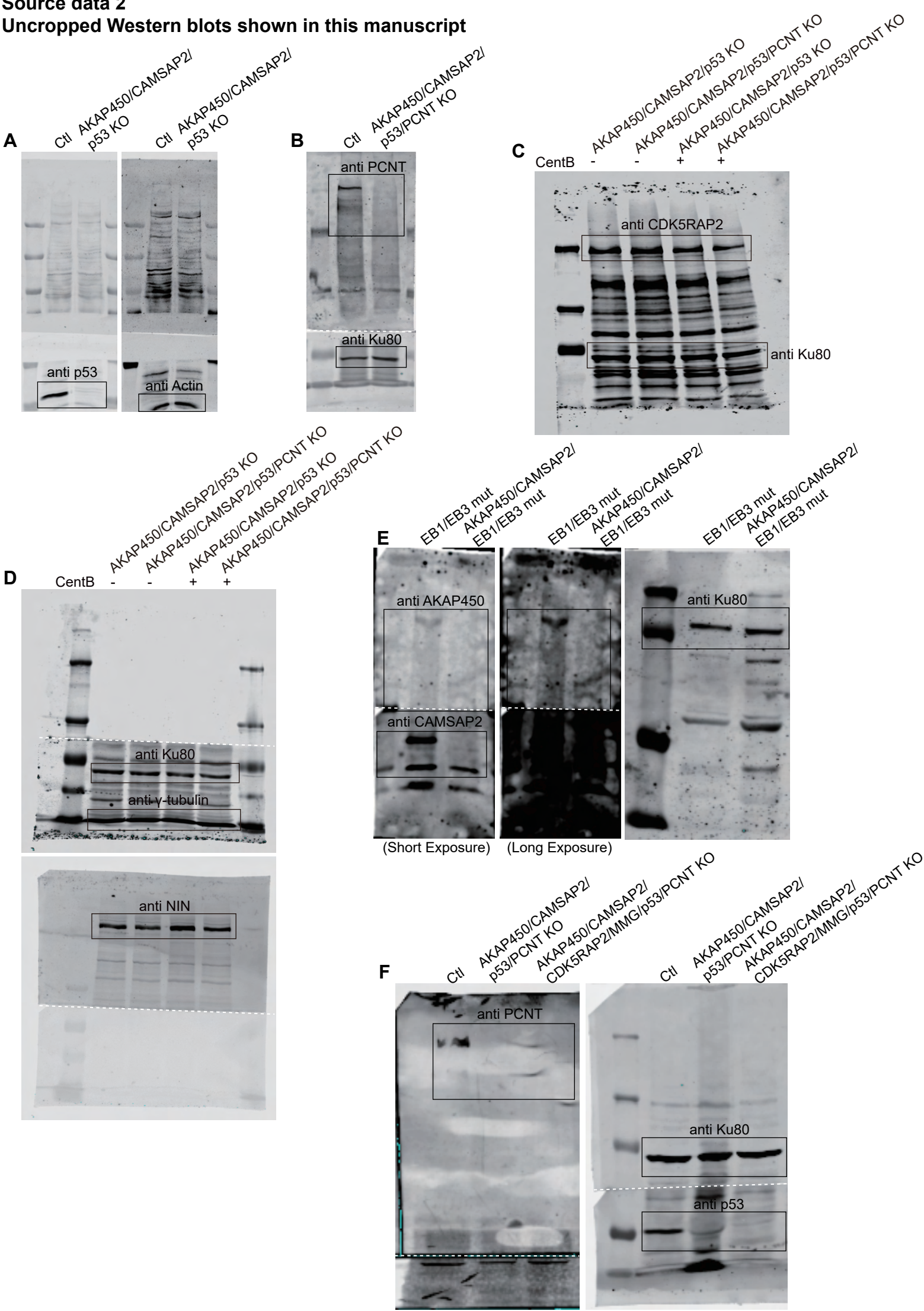

Supplement: Source data 2. — (A) Western blots showing the knockout of p53 from AKAP450/CAMSAP2 knockout cell line shown in Figure 2—figure supplement 2F. (B) Western blots showing the knockout of pericentrin from AKAP450/CAMSAP2/p53 knockout cell line shown in Figure 2—figure supplement 2K. (C-D) Western blots showing expression levels of CDK5RAP2, γ-tub, and ninein (NIN) in control (-CentB) and centrinone-treated AKAP450/CAMSAP2/p53 knockout and AKAP450/CAMSAP2/p53/PCNT knockout cell lines shown in Figure 2—figure supplement 4B. (E) Western blots showing the knockout of AKAP450 and CAMSAP2 from EB1/EB3 mutant RPE1 cell line shown in Figure 2—figure supplement 5B. (F) Western blots showing the knockout of pericentrin in AKAP450/CAMSAP2/CDK5RAP2/MMG/p53 /knockout cell line shown in Figure 7—figure supplement 3G. White dashed lines indicate where the blots were cut before incubation. [file elife-77892-data2.pdf]
